# Invisible Wounds: A Systematic Review of Domestic Violence Against Women

**DOI:** 10.3390/healthcare14040465

**Published:** 2026-02-12

**Authors:** Sorin Deacu, Miruna Cristian, Sabina Ioana Popa, Radu Adrian Nitu, Stefan Pricop

**Affiliations:** 1 Department of Forensic Medicine, County Clinical Emergency Hospital of Constanta, 900591 Constanta, Romania; sorin.deacu@365.univ-ovidius.ro (S.D.); pricop.stefan@ymail.com (S.P.); 2 Faculty of Medicine, Ovidius University, 900470 Constanta, Romania; popasabina_2008@yahoo.com (S.I.P.); radu_nitzu@yahoo.com (R.A.N.); 3 Center for Research and Development of the Morphological and Genetic Studies of Malignant Pathology—CEDMOG, “Ovidius” University, 900591 Constanța, Romania; 4 Department of Cardiovascular Surgery, County Clinical Emergency Hospital of Constanta, 900591 Constanta, Romania

**Keywords:** intimate partner violence, domestic violence, women’s health, mental health, PTSD, depression, gender-based violence

## Abstract

Background: Intimate partner violence (IPV) represents a major global public health concern with profound psychological and social consequences for women. This review synthesizes contemporary evidence (2020–2025) on IPV prevalence, mental health outcomes, and healthcare implications among female populations worldwide. Methods: 18 peer-reviewed studies, encompassing approximately 62,000 women across various countries, were analyzed for study design, sample characteristics, IPV prevalence, and associated outcomes. Results: IPV prevalence varied widely across studies, ranging from 15% in population-based antenatal samples to over 85% among incarcerated or trauma-exposed groups. Across studies reporting mental health outcomes, depression prevalence ranged from 20% to over 50%, while PTSD prevalence ranged from approximately 30% to 70%, depending on measurement tools and population characteristics. No pooled estimates were calculated. IPV survivors showed higher emergency department use (2.6-fold), inpatient admissions (2.2-fold), and healthcare costs (2.2-fold) compared with non-exposed women. Emerging interventions, such as digital safety programs, behavioral antenatal packages, and validated screening tools, demonstrated encouraging effectiveness. Conclusions: IPV remains widespread and linked to psychological distress and elevated healthcare burden. Integration of routine screening, trauma-informed mental health services, and multisectoral prevention frameworks is essential to mitigate its enduring impact on women’s health and well-being.

## 1. Introduction

Intimate partner violence against women is a widespread public health crisis with severe psychological and healthcare consequences, and it is a grave violation of human rights, deeply rooted in systemic gender inequality. According to a fact sheet of the World Health Organization (WHO) (2024), based on 2018 global data synthesis (2000–2018) (uncertainty interval 95%: 26–34%), approximately one in three women globally, around 30%, will experience physical and/or sexual violence in their lifetime, most often at the hands of intimate partners [1]. This staggering figure reflects a global public health problem, transcending cultural, economic, and geographical boundaries. While intimate partner violence and sexual violence are the most frequently reported forms, other manifestations, such as psychological abuse, coercive control, and non-partner sexual assault, compound the breadth and severity of the issue [2].

Reliable data, drawn from population-based surveys across 161 countries between 2000 and 2018, indicate that over a quarter (25–33%) of ever-partnered women aged 15–49 report having experienced physical or sexual violence by an intimate partner during their lifetime [3]. Regional disparities exist as prevalence rates range from around 20% in the Western Pacific, Europe, and high-income countries to 33% in Africa and South-East Asia [1,4]. The WHO further emphasizes that intimate partners are responsible for as many as up to 38% of female homicides are perpetrated by intimate partners, highlighting the lethal consequences of IPV [1].

The consequences of violence against women extend far beyond physical harm. Survivors frequently endure short- and long-term physical injuries, mental health disorders such as depression and PTSD, reproductive health issues, including unintended pregnancies, unsafe abortions, and sexually transmitted infections, and detrimental impacts on children exposed to such violence [5]. Economically and socially, the repercussions ripple outward, affecting not only victims but their families, communities, and the broader society [6].

Root causes are multifaceted, spanning individual, relational, community, and societal levels. Key drivers include unequal gender norms, low education levels, alcohol misuse, exposure to childhood violence, harmful masculine attitudes, and socioeconomic marginalization [7,8].

Women aged 30–39 are often associated with both heightened vulnerability to partner violence and increasing social and economic autonomy, which may empower women to exit violent relationships [9,10]. While divorce is not a direct measure of IPV, its age and gender distribution may signal underlying patterns of abuse that require further exploration [11].

Recognizing this complexity, the WHO-led “RESPECT women” framework outlines seven evidence-based strategies: strengthening relationship skills, empowering women, ensuring services, reducing poverty, creating enabling environments, preventing child and adolescent abuse, and transforming attitudes and norms [12]. In response to this crisis, the World Health Assembly endorsed a global plan of action in 2016 (see Table 1), aimed at integrating violence prevention and response into health systems, supported by population surveillance and multisectoral collaboration [13].

The health sector today is positioned as both a frontline responder, providing care, early identification, and referrals, and as a powerful advocate for legal reform, gender equality, and societal transformation.

This article aimed to critically review the global prevalence of domestic violence against women, to synthesize risk factors, and to appraise prevention frameworks (RESPECT), and it highlights the pivotal roles of policy, health systems, and societal change in combatting this enduring global crisis.

## 2. Materials and Methods

### 2.1. Eligibility Criteria

This review included studies published in peer-reviewed journals between 1 January 2020, and 30 June 2025. Eligible studies met the following inclusion criteria: (1) full-text availability in English, (2) observational or multicenter study design, (3) focus on intimate partner violence or domestic violence against women and adolescent girls, and (4) assessment of health-related outcomes, particularly mental health indicators. Studies involving male-only populations were excluded. For mixed-sex studies, only those providing sex-disaggregated estimates for women were eligible. Mixed-sex studies without separate reporting for women were excluded at the full-text review stage.

We focused on January 2020–June 2025 to capture the most recent post-2018 evidence syntheses and contemporary health-system practices (screening tools, digital interventions, and administrative-data studies).

### 2.2. Information Sources

The primary databases used for this literature retrieval was PubMed, Scopus and Web of Science, which provide coverage of biomedical and public health literature, including epidemiological and clinical studies relevant to intimate partner violence. Searches were conducted up to 30 June 2025, and included only published, full-text, peer-reviewed articles.

### 2.3. Search Strategy

A structured search string using Medical Subject Headings (MeSH) [14] and keywords was applied in our search.

PubMed (MeSH + Keywords): “Intimate Partner Violence” [MeSH] OR “Domestic Violence” [MeSH] OR “violence against women” Filters applied: English, free full text, publication date 1 January 2020–30 June 2025, humans only, adults 19+, randomized controlled trials.

Scopus (MeSH + Keywords): TITLE-ABS-KEY (“intimate partner violence” OR “domestic violence” OR “violence against women”) AND (LIMIT-TO (DOCTYPE, “ar”)) AND (LIMIT-TO (LANGUAGE, “English”)) AND (PUBYEAR > 2020).

Web of Science (MeSH + Keywords): TS = (“intimate partner violence” OR “domestic violence” OR “violence against women”) AND TS = (“mental health” OR depression OR anxiety OR PTSD OR trauma) AND TS = (observational OR “cross-sectional” OR cohort OR multicenter). Filters: English; document type: Article; timespan: 2020–2025.

Database-specific filters were applied pragmatically and no study was excluded solely based on age or design. The final study selection reflects eligibility screening rather than search filter restrictions.

All retrieved citations were imported into EndNote X9 (version X9.3.3 (Build 13966), Clarivate Plc, Philadelphia, PA, USA and London, UK), where automatic duplicate detection was performed using author–title–year matching. Remaining duplicates were identified through manual cross-checking of titles and abstracts by two independent reviewers. This review was registered with Open Science Framework—OSF (Center for Open Science (COS), Charlottesville, VA, USA).

### 2.4. Selection Process

Screening followed PRISMA 2020 guidelines [15]. Two reviewers independently screened all titles and abstracts using a pre-piloted eligibility form. Full texts of potentially eligible studies were retrieved and evaluated independently by both reviewers. Disagreements at either stage were resolved through discussion; a third reviewer adjudicated unresolved conflicts. Screening and extraction were conducted using Microsoft Excel (version 16.0, Microsoft Corporation, Washington, USA), which maintained audit trails for reviewer decisions.

A total of 2979 records were identified across all databases. After removal of 887 duplicate records, 2110 unique citations remained. Following the exclusion of 715 records that were not relevant to the scope of the review, 1395 titles and abstracts were screened. Of these, 1130 records were excluded based on title and abstract assessment, leaving 265 articles for full-text evaluation. Full texts could not be retrieved for 203 studies, resulting in 62 articles assessed for eligibility. Of these, 44 were excluded due to an inappropriate setting or lack of relevance to the research question. Ultimately, 18 studies met all inclusion criteria and were included in the final analysis (see Supplementary Material S1).

Studies are framed as contextual or upstream contributors that inform prevention potential and sociocultural drivers of IPV, rather than as sources of direct health outcome estimates.

### 2.5. Data Collection Process

Data extraction was performed using a standardized template. The following information was retrieved: authors, year, country, study design, sample size, age, population characteristics, type of violence studied, key findings, and study limitations. One reviewer extracted the data and a second verified accuracy. Mental health outcomes were extracted and classified according to the method of assessment used in each study. Outcomes measured using validated psychometric instruments included depressive symptoms, anxiety, PTSD, stress, and related psychological constructs assessed with standardized tools such as the Patient Health Questionnaire-9 (PHQ-9), PTSD Checklist (PCL-5 or PCL-C), Generalized Anxiety Disorder-7 (GAD-7), Beck Depression Inventory-II (BDI-II), Escala de Gravedad de Síntomas del Trastorno de Estrés Postraumático (EGEP-5), and validated quality-of-life or stress scales. In contrast, several studies assessed mental health outcomes using indirect or administratively derived indicators, including diagnostic codes from insurance claims or electronic health records, routine clinical documentation in emergency or primary care settings, biological proxy indicators, or algorithm-based identification systems applied to routinely collected healthcare data. Given the differing levels of diagnostic precision, these two categories of mental health assessment were analyzed and interpreted separately.

### 2.6. Data Items

**Figure 1:**
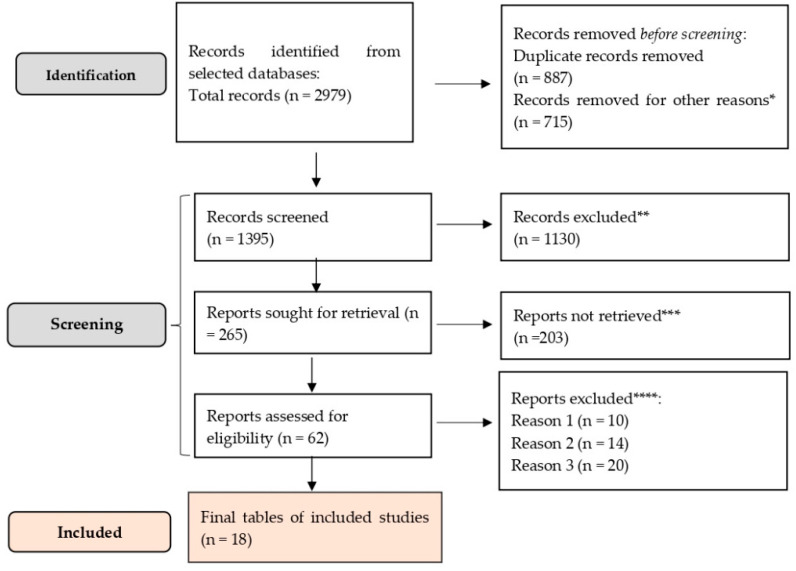
PRISMA framework. * studies are not relevant for the present review. ** non RCT, wrong population. *** unable to find the full text of the study. **** Reason 1—study on animals/Reason 2—wrong setting/Reason 3—research question not relevant.

Key variables extracted included: study location, population demographics, IPV type (physical, sexual, emotional), mental health outcomes (depression, PTSD, anxiety), prevalence rates, and statistical associations (odds ratios, adjusted prevalence ratios, etc.). The flow diagram below (see Figure 1) illustrates the selection process for studies included in this review based on the PRISMA 2020 guidelines [15].

### 2.7. Risk of Bias

The methodological quality and risk of bias of the included studies were assessed using design-appropriate tools recommended for systematic reviews of health research. Randomized controlled trials and randomized experiments were evaluated with the revised Cochrane Risk of Bias tool for randomized trials (RoB 2—Cochrane, UK) [16]. Non-randomized cohort and comparative observational studies were appraised using the ROBINS-I tool (Risk Of Bias In Non-randomized Studies—of Interventions—Cochrane, UK) [16]. Cross-sectional and case–control studies were assessed with the Joanna Briggs Institute (JBI) Critical Appraisal Checklists specific to each design [17].

For all tools, two reviewers independently performed the risk of bias assessment after a calibration exercise on a pilot subset of studies. Discrepancies were resolved through discussion; when consensus could not be achieved, a third reviewer adjudicated. For reporting consistency, tool-specific ratings were harmonized into three overall categories: low risk of bias, some concerns/moderate risk of bias, and high/serious risk of bias.

ROBIS (Risk Of Bias In Systematic Reviews) was not applied, as all included records were primary research studies rather than systematic reviews [16].

### 2.8. Approach to Evidence Synthesis

Because the included studies differed substantially in design (cross-sectional surveys, RCTs, retrospective cohorts), population characteristics, screening instruments, and outcome metrics, pooled statistical synthesis was not feasible, instead context-specific evidence clusters IPV prevalence were reported using non-comparable definitions, and mental health outcomes were measured using heterogeneous scales (clinical diagnosis, or single-item indicators). Given these methodological inconsistencies, any numerical pooling, whether through arithmetic means or meta-analysis, would produce biased or misleading estimates.

Therefore, in line with Cochrane guidance for heterogeneous evidence, we present a narrative synthesis only, reporting study-specific prevalence values, ranges, and descriptive patterns without computing aggregated means.

## 3. Results

Table 2 below synthesize key details from the eighteen peer-reviewed studies examining the prevalence, characteristics, and health impacts of intimate partner violence and related forms of abuse against women.

### 3.1. Overview of Included Studies

This review encompassed 18 original studies published between 2020 and 2025, reflecting the most recent global research on intimate partner violence (IPV) and its multifaceted health and psychosocial consequences among women.

From a methodological standpoint, cross-sectional investigations remained predominant (*n* = 8), typically utilizing community surveys, national health datasets, or clinical records to assess IPV prevalence, risk correlates, and associated health outcomes. Notable examples include the works of Cabrales-Tejeda et al. [18], Charak et al. [22], Daugherty et al. [23], Bentley et al. [26], Chaquila et al. [27], Kelly et al. [32], and Panjaphothiwat et al. [34], each addressing distinct populations such as reproductive-age women, migrant communities, or adolescents. Two additional cross-sectional validation studies, Guiguet-Auclair et al. [25] and Ghafournia and Healey [20], focused on the development and diagnostic performance of IPV screening instruments used in healthcare and emergency settings.

Randomized controlled trials accounted for a substantial proportion of the recent evidence base (*n* = 5), reflecting a growing emphasis on intervention-based research. These included trials evaluating educational or behavioral programs (Agde et al. [28], Barata et al. [29], Mahapatro et al. [33]), trauma-focused psychotherapeutic approaches (Crespo et al. [30]), and online or digital interventions (Ford-Gilboe et al. [24]). The cluster-RCT design adopted by Taft et al. [35] further extended this approach to primary care, assessing domestic violence identification systems within culturally diverse clinical populations.

In contrast, retrospective and data-driven studies (*n* = 2) explored IPV through large-scale administrative or health record datasets. Kishton et al. [19] analyzed over 10,000 cases of documented violence using U.S. insurance claims, while Emezue et al. [9] employed machine learning algorithms applied to electronic health records to predict IPV risk and related mental health outcomes, illustrating a contemporary shift toward data-informed surveillance and predictive analytics.

The studies represent a wide geographical spectrum, covering four continents and 11 countries. North American research contributed the largest share (United States and Canada; *n* = 6) [9,19,21,23,24,29], followed by European investigations (United Kingdom, France, and Spain; *n* = 4) [22,25,26,30]. Studies from Latin America, specifically Mexico and Peru, accounted for two [18,27], while additional contributions originated from Asia (India, Thailand, and Ethiopia; *n* = 3) [28,33,34] and Oceania (Australia; *n* = 1) [35].

Sample sizes varied markedly, reflecting differences in study scope and data sources. The smallest participant group (*n* = 93) was reported by Daugherty et al. [23], who examined executive functioning among shelter-based survivors, whereas the largest sample (*n* = 45,438) derived from Taft et al. [35], a population-level general practice dataset from Australia. Similarly, Chaquila et al. [27] analyzed over 18,000 women from Peru’s national health survey, while Emezue et al. [9] utilized a dataset exceeding one million electronic health records. The populations studied encompassed migrant women accessing support services [26], pregnant women attending antenatal care [33,34], incarcerated or trauma-exposed women [21,30], and adolescents in sub-Saharan Africa [32].

Additional descriptive statistics and extended study-level information are provided in Supplementary Material S2, which offers a more detailed breakdown of sample characteristics, prevalence values, and outcome pattern.

**Figure 2:**
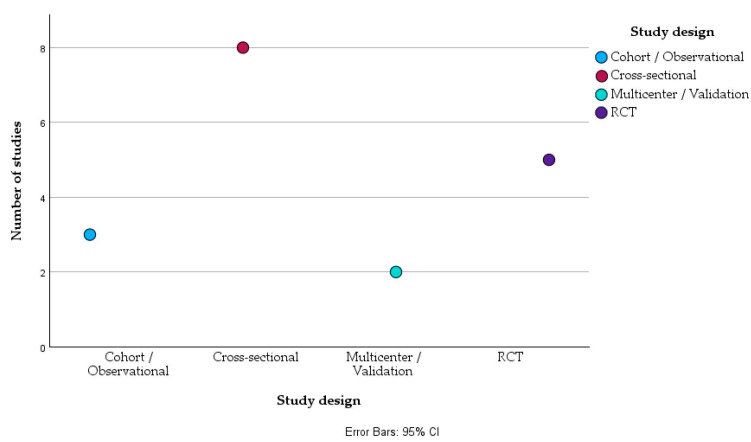
Distribution of selected studies according to their type (Cohort/Observational—3, Cross-sectional—8, Multicenter/Validation—2, RCT—5). All values represented in this figure are derived directly from the study-level numerical data presented in Table 2A,B. No statistical pooling or inferential modeling was applied; the figure illustrates descriptive patterns across heterogeneous studies.

As shown in the top panel (see Figure 2), the most frequent study design among the included articles was cross-sectional (*n* = 8), followed by randomized controlled trials (RCTs) (*n* = 5). Cohort or observational studies, including longitudinal and retrospective analyses, accounted for *n* = 3, while multicenter or validation studies represented *n* = 2.

**Figure 3:**
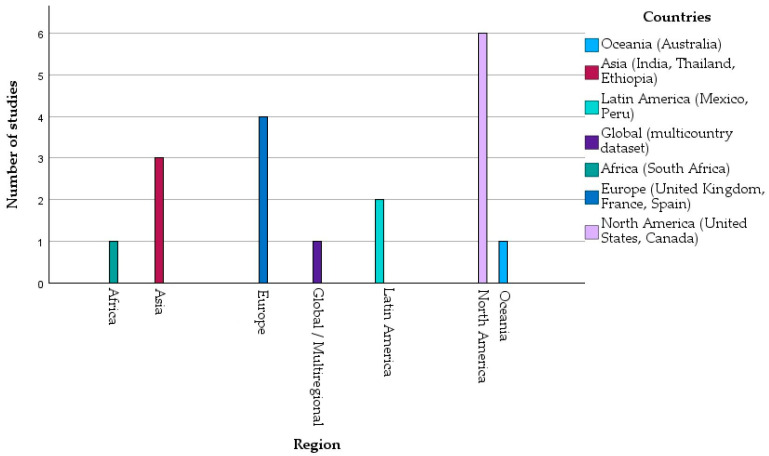
Distribution of selected studies according to their location. All values represented in this figure are derived directly from the study-level numerical data presented in Table 2A,B. No statistical pooling or inferential modeling was applied; the figure illustrates descriptive patterns across heterogeneous studies.

The bottom panel in Figure 3 displays the geographical distribution of the studies. North America contributed the largest number (*n* = 6), followed by Europe (*n* = 4), Asia (*n* = 3), Latin America (*n* = 2), and Oceania (*n* = 1). In addition, one study used a multiregional or global dataset.

### 3.2. Population Characteristics

**Figure 4:**
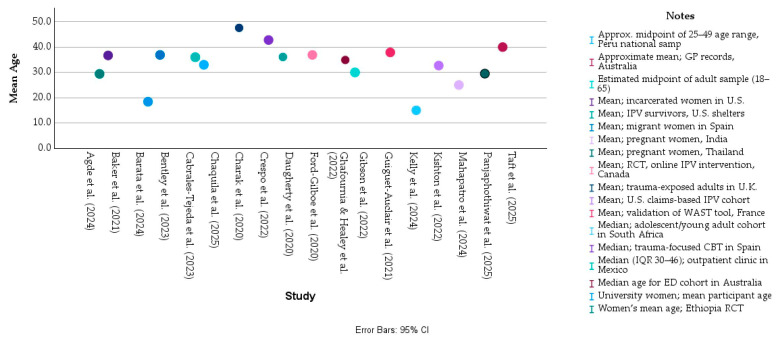
Distribution of age across the selected studies. All values represented in this figure are derived directly from the study-level numerical data presented in Table 2A,B. No statistical pooling or inferential modeling was applied; the figure illustrates descriptive patterns across heterogeneous studies [18,19,20,21,22,23,24,25,26,27,28,29,30,31,32,33,34,35].

The eighteen studies included in this review encompassed a broad and heterogeneous spectrum of female populations affected by intimate partner violence or broader forms of interpersonal and gender-based trauma. Across the literature, participants were predominantly women in their reproductive and early adult years, with reported mean or median ages ranging from the mid-20s to late-40s. As illustrated in the graph below (see Figure 4), the majority of samples centered around women in their thirties, consistent with the demographic profile most frequently affected by IPV globally. The oldest cohort was observed in the UK-based study by Charak et al. [22], which analyzed trauma-exposed adults from a nationally representative panel with a mean age of 47.6 years, while the youngest populations appeared in the Peruvian national survey by Chaquila et al. [27] and the South African adolescent cohort examined by Kelly et al. [32], where participants were primarily in their teens and twenties.

From a sociodemographic standpoint, the reviewed studies addressed diverse and often socially vulnerable populations, encompassing groups defined by socioeconomic disadvantage, migration status, pregnancy, and exposure to trauma or marginalization. For example, Bentley et al. [26] examined migrant women residing in Spain, predominantly of Latin American origin, accessing social support services, and reporting high levels of psychological IPV and comorbid depression and PTSD. In contrast, Baker et al. [21] investigated incarcerated women in the United States undergoing trauma-informed therapy.

At the population level, Chaquila et al. [27] analyzed 18,621 reproductive-age women (15–49 years) using Peru’s national health survey revealing a significant association between IPV and depressive symptoms—notably moderated by household wealth, with the strongest effects observed in both the poorest and wealthiest strata. Clinical and healthcare-based studies also represented a substantial segment of the evidence base. Cabrales-Tejeda et al. [18] examined women attending gynecology outpatient clinics in Mexico, most of whom were married and from low-income backgrounds, demonstrating that domestic violence often persists within apparently stable family units. Similarly, Ghafournia and Healey [20] studied emergency department patients in Australia, identifying frequent recurrent visits and coexisting pregnancy-related presentations as indicators of ongoing IPV risk.

Additionally, several studies targeted pregnant women as a high-risk group, such as Mahapatro et al. [33] in India and Panjaphothiwat et al. [34] in Thailand, both of which found strong links between IPV exposure, mental health burden, and adverse reproductive outcomes. Others, like Agde et al. [28] and Gibson et al. [31], addressed male–female dyads and community-level attitudes, offering insights into how sociocultural norms, education, and alcohol use shape IPV acceptance and prevention potential.

### 3.3. Assessment Method, Prevalence and Patterns of Intimate Partner Violence

The prevalence of intimate partner violence varied markedly across the included studies. Mental health outcomes were assessed using heterogeneous methodologies across the included studies, as detailed in Table 2B. Several studies employed validated psychometric instruments, including PHQ-9, PCL-5, GAD-7, EGEP-5, BDI-II, and standardized quality-of-life measures, enabling synthesis of depression, anxiety, PTSD, and stress-related outcomes [21,22,23,26,27,30]. In contrast, other studies derived mental health information from administrative diagnostic codes or clinical documentation [19,20,35], biological or proxy indicators [32], or did not assess mental health outcomes [18,25,28,29].

To facilitate interpretation, findings are summarized across four major contexts:

(a) Community and general population studies (*n* = 3)

Population-based investigations from Mexico [18], Peru [27], and Ethiopia [31] provided broad estimates of IPV prevalence among women in the general population. Reported rates ranged between 28% and 54%, depending on measurement tools and reporting bias adjustments. In Mexico, Cabrales-Tejeda et al. [18] found that 52% of women attending gynecology clinics had experienced IPV at least once, while the Peruvian national survey [27] identified a similar prevalence of 54% among reproductive-age women, with emotional and physical abuse predominating. In Ethiopia, Gibson et al. [31] revealed that support for physical IPV reached 28% under indirect questioning versus 18% under direct self-report.

(b) Clinical and healthcare settings (*n* = 4)

Studies conducted in hospital, antenatal, and emergency department contexts reported some of the highest IPV detection rates. Ghafournia and Healey [20] observed IPV in approximately 60% of women presenting to an Australian emergency department, with particularly elevated rates among pregnant and Indigenous patients. Similarly, Panjaphothiwat et al. [34] identified an IPV prevalence of 15.5% among pregnant women in Thailand during the COVID-19 pandemic, while Mahapatro et al. [33] demonstrated that a behavioral intervention in New Delhi led to a 90% reduction in severe IPV cases post-intervention. The French validation study by Guiguet-Auclair et al. [25] reported that 52% of women screened positive for IPV using the WAST-F tool.

(c) High-risk and vulnerable populations (*n* = 5)

Elevated IPV prevalence was reported among marginalized or trauma-affected groups. Bentley et al. [26] found that 78% of migrant women in Spain experienced psychological, physical, or sexual IPV, with comorbid depression and PTSD common among those exposed to multiple abuse types. Among incarcerated women in the U.S., Baker et al. [21] reported that all participants had experienced interpersonal trauma, primarily sexual violence, while Crespo et al. [30] confirmed near-universal IPV exposure in their sample of Spanish women enrolled in trauma-focused therapy. Similarly, Ford-Gilboe et al. [24] and Kishton et al. [19] reported 100% IPV exposure, as both studies specifically recruited women with ongoing or documented histories of abuse for intervention or healthcare utilization analyses.

(d) Adolescent and young adult populations (*n* = 2)

Two studies focused on younger cohorts. Barata et al. [29] observed a 54% reduction in IPV incidence among first-year university women who completed a sexual assault resistance education program in Canada, while Kelly et al. [32] identified distinct IPV trajectory groups among South African adolescent girls and young women, with 26.7% following a high-risk trajectory characterized by recurrent exposure and associated inflammatory stress markers.

**Figure 5:**
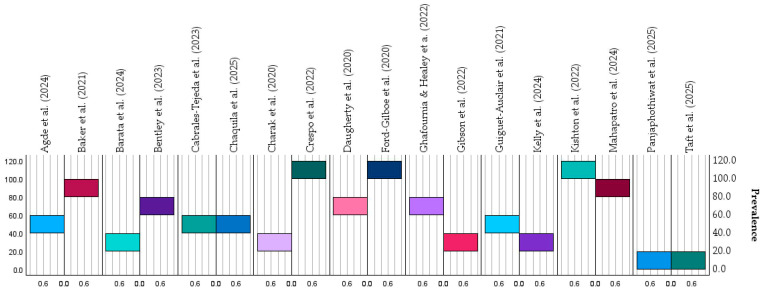
Prevalence patterns of IPV by type, including physical, sexual, psychological, and coercive control [18,19,20,21,22,23,24,25,26,27,28,29,30,31,32,33,34,35]. Values are expressed as percentages of participants reporting each form of IPV in the included studies. They are derived directly from the study-level numerical data presented in Table 2A,B. No statistical pooling or inferential modeling was applied; the figure illustrates descriptive patterns across heterogeneous studies. The colors are for visual purposes only.

The prevalence of IPV across all studies ranged from 15% to 100%, depending on population type, measurement method, and inclusion criteria (see Figure 5). The highest prevalence levels were observed among clinical and high-risk groups, particularly those recruited from trauma, emergency, or support services, whereas national or community-based surveys tended to yield moderate but consistent prevalence estimates (40–55%).

Subgroup-specific investigations revealed consistently high levels of IPV and related trauma exposure across vulnerable populations. Baker et al. [21] reported an 85% prevalence of cumulative interpersonal trauma among incarcerated women in the United States, with the majority of experiences tracing back to childhood and adolescence, underscoring the cyclical nature of victimization across the lifespan. Similarly, Ghafournia and Healey [20] documented IPV prevalence rates approaching 60% among women presenting to emergency departments in Australia, identifying particularly high risk among pregnant and Indigenous patients, where recurrent visits often signaled ongoing abuse.

In Spain, Crespo et al. [30] observed that nearly all participants in trauma-focused cognitive-behavioral therapy had a history of IPV, while Bentley et al. [26] found that 78% of migrant women accessing support services had endured psychological, physical, or sexual abuse, frequently accompanied by depression and PTSD. Complementing these findings, Daugherty et al. [23] reported that over 70% of IPV survivors recruited from shelters and online platforms experienced repeated or persistent abuse, which was correlated with impairments in perceived executive functioning, particularly difficulties with concentration, information processing, and attention.

### 3.4. Mental Health and Health-Related Outcomes

Mental health outcomes emerged as a central theme across the majority of included studies, with 13 of the 18 investigations explicitly examining the psychological and neurocognitive sequelae of IPV. The most commonly assessed outcomes were depression and post-traumatic stress disorder, followed by anxiety, suicidality, and executive dysfunction. Within the selected studies of this review, validated psychometric tools such as the Patient Health Questionnaire-9 (PHQ-9), the PTSD Checklist-Civilian Version (PCL-C), and structured diagnostic interviews were frequently utilized to quantify symptom severity and diagnostic thresholds.

Among vulnerable and marginalized groups, the burden of mental distress was striking. Bentley et al. [26] reported that 53.5% of migrant women in Spain met the threshold for clinically significant depression, while 34.6% screened positive for PTSD. Comorbid exposure to multiple IPV forms (psychological, physical, and sexual) was associated with markedly worse psychiatric outcomes than exposure to a single abuse type. Similarly, in the United Kingdom, Charak et al. [22] identified that polyvictimized women, those exposed to several types of interpersonal violence, had 9-fold higher odds of depression, 12-fold higher odds of anxiety, and 33-fold higher odds of PTSD, as well as significantly greater suicidality compared with limited-victimization groups.

In institutional contexts, Baker et al. [21] observed alarmingly high rates of PTSD, anxiety, and suicidal ideation among incarcerated women in the United States, nearly all of whom reported cumulative trauma beginning in childhood. Crespo et al. [30] found that among Spanish survivors of intimate partner and sexual violence undergoing trauma-focused cognitive behavioral therapy, high baseline levels of anxiety and posttraumatic cognitions predicted treatment dropout, particularly among younger and migrant participants.

Population-level data reinforced these associations. Chaquila et al. [27] demonstrated a robust link between IPV and depressive symptoms in over 18,000 Peruvian women of reproductive age, with the association persisting across all socioeconomic strata and paradoxically strongest among the wealthiest quintiles. Likewise, Kelly et al. [32] found that South African adolescents in the high IPV trajectory group exhibited elevated systemic inflammation (increased C-reactive protein).

### 3.5. Healthcare Utilization and System Burden

Patterns of healthcare use further reflected the substantial mental health burden associated with IPV. Kishton et al. [19], analyzing over 10,000 U.S. insurance claims, identified significantly higher emergency department and inpatient service use, increased mental health and substance use diagnoses, and greater healthcare costs among women with documented violence compared to controls. Similarly, Taft et al. [35] demonstrated the limitations of existing clinical identification systems, with domestic violence recorded in only 0.58% of women’s medical records.

Beyond formal healthcare encounters, several studies highlighted patterns suggestive of recurrent, fragmented, or delayed engagement with health services among women exposed to IPV. Ghafournia and Healey [20] reported high rates of repeat emergency department presentations among women affected by domestic violence and sexual assault, often accompanied by mental health symptoms and pregnancy-related concerns. In clinical and support-seeking populations, elevated psychological morbidity was frequently associated with increased service contact rather than coordinated care pathways [21,26].

### 3.6. Intervention and Screening-Related Evidence

Evidence related to interventions and screening strategies was reported in a limited but methodologically diverse subset of the included studies. Several randomized controlled trials evaluated structured prevention or support interventions and demonstrated favorable outcomes. Ford-Gilboe et al. [24] reported sustained improvements in IPV-related safety and mental health indicators following a tailored online intervention for women experiencing violence. Behavioral and educational interventions delivered in antenatal or community settings were also associated with reductions in IPV severity or improvements in quality of life [29,33]. In parallel, screening-focused studies emphasized both the potential and limitations of current identification approaches. Guiguet-Auclair et al. [25] demonstrated high diagnostic accuracy of the Women Abuse Screening Tool in routine clinical settings, whereas Taft et al. [35] highlighted substantial under-identification of IPV in primary care records.

**Figure 6:**
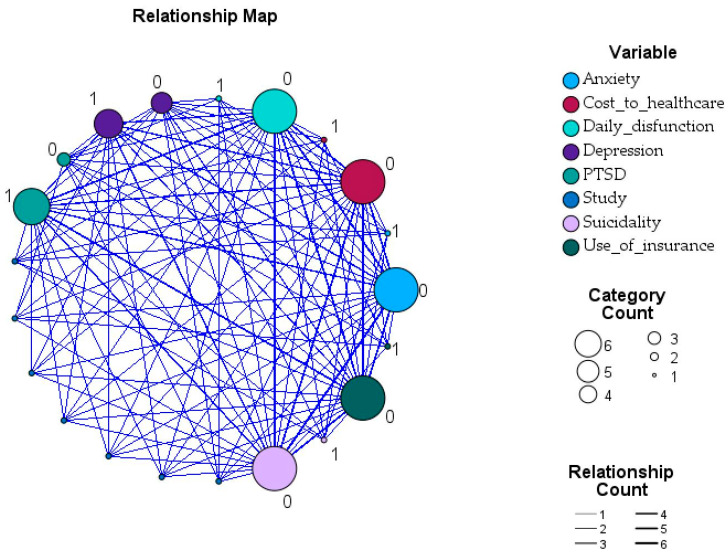
Network visualization of reported mental health and healthcare outcomes in IPV studies. All values represented in this figure are derived directly from the study-level numerical data presented in Supplemental Material, derived from Table 2A,B. No statistical pooling or inferential modeling was applied; the figure illustrates descriptive patterns across heterogeneous studies.

To further explore how psychological and healthcare-related variables interact within the evidence base, we computed pairwise Pearson correlations [36] among the key outcomes reported across the studies that actually reported those variables numerically (Supplementary Material S3). The correlation matrix below summarizes the direction and relative strength of associations between PTSD, depression, anxiety, suicidality, daily dysfunction, and two indicators of healthcare utilization (cost to healthcare and use of insurance). Although these correlations are based on aggregated study-level indicators and should not be interpreted as individual-level effects, they offer a descriptive overview of how frequently these outcomes co-occur in the literature and provide the foundation for the network visualization presented in Figure 6.

The strongest positive correlation was observed between anxiety and suicidality (r = 1.00, *p* < 0.001), indicating that studies reporting anxiety symptoms almost invariably reported suicidality as well. PTSD showed weak positive correlations with depression, anxiety, and suicidality, though none reached statistical significance. Daily dysfunction demonstrated moderate negative correlations with PTSD (r = −0.65) and use of insurance (r = −0.65). Depression showed consistently negative correlations with anxiety, suicidality, and daily dysfunction, though these associations were modest and non-significant.

This relationship map visualizes the frequency and co-occurrence of major mental health and healthcare-related outcomes reported across the studies included in this review. It is descriptive and illustrative, not inferential, and its purpose is limited to visualizing co-occurrence of outcomes across studies rather than estimating associations.

Each node represents a specific outcome variable, such as depression, PTSD, anxiety, suicidality, daily or executive dysfunction, and healthcare utilization measures (e.g., costs, insurance use). The node size corresponds to how frequently that outcome was reported across studies, while the thickness of connecting lines reflects the strength of co-occurrence, indicating how often two variables were assessed within the same study.

As shown, depression and PTSD form the most prominent hubs within the network, representing the core psychological sequelae most consistently evaluated in IPV-related research. These outcomes were interconnected with anxiety, suggesting overlapping symptom clusters and shared measurement frameworks. Suicidality and executive or daily dysfunction appeared less frequently but maintained close associations with PTSD and depression, underscoring the cascading cognitive and emotional impact of chronic abuse. Meanwhile, healthcare utilization indicators, including cost-to-healthcare and use-of-insurance, occupied a peripheral but important position, linking psychological morbidity with tangible system-level consequences.

These visualizations are descriptive and illustrative and do not represent individual-level effects, pooled estimates, or statistically meaningful associations.

### 3.7. Risk of Bias in Included Studies

The methodological quality of the included studies was mixed (Table 3). Three studies were judged to have low risk of bias, three were rated as high/serious risk of bias, and the remaining twelve presented some concerns or moderate risk of bias. The assessments were conducted for transparency but did not influence weighting of conclusions.

Among randomized controlled trials and randomized experiments appraised with RoB 2, most were rated as having *some concerns*, primarily due to selective or self-selected samples (e.g., university students or women with internet access), limited blinding, and reliance on self-reported outcomes for IPV and mental health. The RCTs by Barata et al. [29] and Gibson et al. [31] demonstrated generally robust randomization and outcome assessment procedures and were therefore classified as low risk of bias, whereas Crespo et al. [30] was judged at high risk due to substantial early dropout, small sample size, and single-therapist delivery of the intervention.

Observational and cross-sectional designs assessed with JBI checklists typically raised concerns related to sampling and measurement. Many studies relied on convenience or clinic-based samples (e.g., gynecology outpatients, emergency department attendees, incarcerated women, or support-seeking migrants), which may limit external validity and introduce selection bias [18,20,21,26]. In addition, IPV and mental health outcomes were often based on retrospective self-report, with limited adjustment for potential confounders, raising the possibility of recall and information bias [21,22,23,26,27,34]. These limitations resulted in “some concerns” ratings for the majority of cross-sectional studies.

Two comparative cohort analyses, Kishton et al. [19] and Kelly et al. [32], were evaluated with ROBINS-I. Both were judged to have moderate risk of bias due to residual confounding, incomplete control for socioeconomic and contextual factors, and potential misclassification of exposure (e.g., reliance on administrative codes or repeated self-report over time).

Studies rated as high or serious risk of bias predominantly involved small, highly selected samples, retrospective assessment of complex trauma histories, and limited control of confounding variables. This applied particularly to research conducted among incarcerated women and trauma-focused treatment samples, where cumulative trauma, comorbidities, and attrition could not be fully disentangled from measured outcomes [21,23,30].

Despite these methodological constraints, a consistent pattern emerged across low- and moderate-risk studies: intimate partner violence was associated with elevated depression, PTSD, anxiety, and, in some cohorts, increased healthcare utilization and systemic inflammation [19,22,26,27,32].

For reporting consistency and to facilitate comparison across heterogeneous designs, all qualitative judgments were additionally converted into numerical codes as follows: 1 = low risk, 2 = some concerns, 3 = moderate risk, and 4 = high/serious risk of bias. These codes enabled standardized synthesis across RoB 2, ROBINS-I, and JBI tools. The final harmonized ratings for all 18 studies are presented in Table 3.

**Figure 7:**
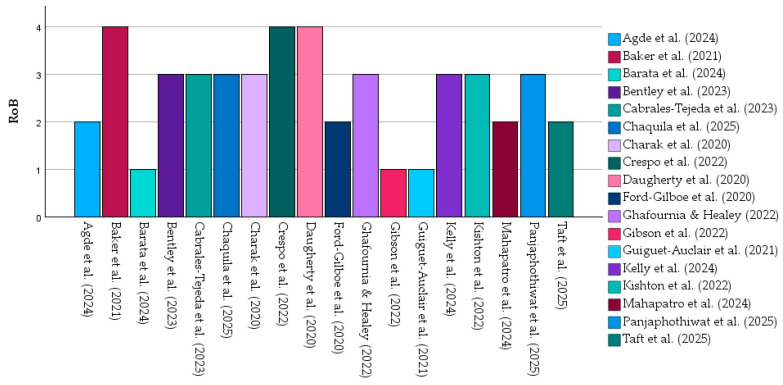
Numerical risk of bias scores for the 18 included studies [18,19,20,21,22,23,24,25,26,27,28,29,30,31,32,33,34,35]. All values represented in this figure are derived directly from the study-level numerical data presented in Table 2A,B. No statistical pooling or inferential modeling was applied; the figure illustrates descriptive patterns across heterogeneous studies.

The distribution of numerical risk-of-bias ratings across the 18 included studies is illustrated in Figure 7. Each bar represents an individual study and its corresponding risk.

As shown, most studies fell within the moderate-risk category, reflecting limitations related to sampling methods, self-reported outcomes, and cross-sectional designs. Only a small number of trials demonstrated low risk of bias, while three studies exhibited high risk, primarily due to significant attrition, retrospective trauma measurement, or highly selected populations.

Next, we conducted a narrative sensitivity analysis by comparing results from studies with low or moderate risk of bias to those derived mainly from high-risk studies (Table 4).

Interpretation of study findings was guided by methodological quality and risk-of-bias assessment. Results derived from studies assessed as having low or moderate risk of bias were considered more reliable and constitute the primary basis of the descriptive synthesis. In contrast, findings originating from studies with high risk of bias, including those involving small sample sizes, highly selected populations, or retrospective trauma assessment, were interpreted with caution and are presented as exploratory and hypothesis-generating rather than definitive.

Several core conclusions are supported primarily by studies with low or moderate risk of bias. In contrast, conclusions related to extreme prevalence estimates, suicidality severity, and cognitive dysfunction are driven mainly by high-risk or highly selected samples and should therefore be interpreted cautiously as hypothesis-generating rather than definitive evidence.

## 4. Discussion

Consistent with prior research, our review found that IPV remains highly prevalent across diverse settings and populations. The included studies are regarded as contextual evidence that report IPV rates ranging from 33% to 100%, with highest prevalence observed among incarcerated women [21], migrant populations [26], and clinical samples selected for IPV exposure [19,24]. These rates echo the global burden reported by the World Health Organization [1], which estimates that one in three women worldwide experiences physical or sexual violence in her lifetime. The study by Ghafournia and Healey [20] adds to this picture by highlighting the intersection of IPV with pregnancy and Indigenous status in emergency department presentations, an area previously underrepresented in the literature. Interpretations drawn from these studies are explicitly limited to sociocultural context and prevention relevance

From a biological perspective, exposure to chronic stressors has been shown to induce measurable tissue- and system-level changes. Experimental studies demonstrate that biological structures respond adaptively to sustained external stimuli, as evidenced by histomorphometry alterations following electrical or ultrasound stimulation [36].

In analyzing the evidence, no pooled estimates were calculated due to the substantial heterogeneity across study designs, populations, and outcome definitions. Consequently, our conclusions rely on ranges and study-level findings rather than any form of statistical aggregation.

Our synthesis also supports prior findings on the significant mental health burden associated with IPV. Similar to Charak et al. [22], White et al. [37] demonstrates a strong association between polyvictimization and poor mental health outcomes.

Procaccia’s et al. [38] cohort of women with histories of IPV reported significant depression and PTSD symptoms, which supports the work of Daugherty et al. [23] and Bentley et al. [26], both included in our analysis.

Similar to the findings of Baker et al. [21], Augsburger et al. [39] reported that 43.3% of the incarcerated women included in their study reported mental health problems; the most frequent self-identified diagnoses were depressive (20.0%) and anxiety (20.0%) disorders.

Comparable to Kishton et al. [19], Davidov et al. [40] also used healthcare data and cost burden to assess IPV. Thus, the researchers state that women enrolled in their study seeking care in the Emergency Departments were more likely than those at Urgent Care clinics to report lifetime physical or sexual IPV, tobacco use, drug abuse, anxiety, and depression.

At the policy level, these intervention strategies align directly with global frameworks prioritizing violence prevention and gender equity. The recommendations emerging from this review are consistent with Sustainable Development Goal 5 (Gender Equality), specifically Target 5.2, which calls for the elimination of all forms of violence against women in public and private spheres [41]. Furthermore, the proposed actions reinforce the WHO Global Plan of Action 2025, which urges Member States to strengthen health-sector capacity for early identification, clinical management, data documentation, and coordinated multisectoral response to violence against women [42].

In accordance with Cochrane guidance for the synthesis of heterogeneous evidence, quantitative pooling was not undertaken in this review. The included studies differed substantially with respect to study design, populations, definitions of intimate partner violence, measurement instruments, and outcome reporting, rendering meta-analytic aggregation inappropriate and potentially misleading. Consequently, the discussion is based on narrative synthesis, with conclusions drawn from study-level ranges, descriptive patterns, and the consistency of associations observed across independent investigations.

Conclusions were primarily informed by studies with low or moderate risk of bias, including population-based surveys, longitudinal cohorts, and randomized controlled trials. Evidence derived from high-risk or highly selected samples, such as incarcerated populations or treatment-seeking survivors, was interpreted conservatively and is presented as exploratory, serving to generate hypotheses and inform future research rather than to support generalized or causal conclusions.

In contrast, findings derived from studies judged to be at high or serious risk of bias were interpreted with caution and used mainly to illustrate potential trends rather than to substantiate definitive conclusions.

### Strengths and Limitations

This review has several strengths. It synthesizes evidence from diverse populations across multiple regions, including community-based surveys, clinical samples, and high-risk subgroups such as incarcerated or migrant women. The review also highlights mental health outcomes and healthcare utilization, areas that are often underexplored in IPV research.

Nevertheless, important limitations must be acknowledged. First, the eligibility criteria excluded male-only studies and required women-specific estimates; as a result, mixed-sex studies without sex-disaggregated data were omitted, which may limit generalizability. Secondly, considerable heterogeneity was observed across measurement tools and outcome definitions, including variations in IPV screening instruments, mental health scales, and healthcare-use indicators. Moreover, the relatively small number of eligible studies (*n* = 18) restricts the robustness of pooled interpretation. Finally, only English-language studies were included, which may introduce language bias and result in the exclusion of relevant research conducted in low- and middle-income regions where IPV prevalence and reporting patterns may differ substantially.

Despite these constraints, the findings provide a timely and rigorous synthesis of IPV prevalence and outcomes in women across different contexts. Thus, the conclusions from our included studies corroborate and extend the literature by reaffirming IPV’s widespread prevalence, documenting its profound mental health consequences, highlighting underexplored populations, and pointing to both systemic burden and intervention pathways. Continued research is needed to tailor responses to local contexts and high-risk groups.

## 5. Conclusions

### 5.1. Evidence-Based Conclusions

Across the 18 included studies, IPV prevalence ranged from 15% in population-based samples to nearly 100% in high-risk clinical or trauma-exposed groups. This wide range reflects heterogeneity in populations, measurement tools, and study designs, rather than differences in true underlying prevalence. Depression and PTSD emerged as the most consistently reported psychological outcomes, generally affecting one-third to two-thirds of women exposed to IPV. Anxiety, suicidality, executive dysfunction, and elevated systemic inflammation were also documented in several studies, particularly among survivors with cumulative or polyvictimization histories.

Healthcare-related impacts were evident in data-driven studies: IPV survivors demonstrated increased emergency department use (2.6×), higher hospitalization rates (2.2×), and greater healthcare expenditures (2.2×). At the same time, under-detection of IPV within medical systems was notable, with documentation rates as low as 0.58% in primary care records. These findings are grounded exclusively in the empirical evidence synthesized from the included studies and do not represent extrapolated or pooled effects.

### 5.2. Policy and Practice Implications

Although the included studies do not evaluate national policies directly, their findings highlight several areas of practical relevance. Routine screening in clinical settings, especially emergency departments, antenatal care, and primary care, appears highly warranted given the high IPV burden observed in these contexts.

## Figures and Tables

**Table 1:** Magnitude of interpersonal violence across the life-course [13].

Age Group	Key Statistics
Early and Middle Childhood (0–9 years)	- Over 67 million girls (aged 20–24 years) were married before the age of 18. - >125 million girls in 29 countries have undergone female genital mutilation (FGM) - 20% of girls and 5–10% of boys experience child sexual abuse. - 25% of children experience physical violence and 36% emotional violence. - 42% of girls and 37% of boys have been bullied by peers in the past 30 days.
Adolescence (10–19 years)	- 1 in 3 girls (aged 15–49 years) experienced physical and/or sexual violence by an intimate partner. - An estimated 7% of girls have been sexually assaulted by someone other than a partner since age 15. - 1 in 2 children and 1 in 4 have experienced a physical fight with peers in the past year.
Youth (20–24 years)	- An estimated 11.4 million women and girls have been trafficked.
Adult (25–49 years)	- 38% of homicides against women and 6% of homicides against men are perpetrated by intimate partners. - Millions of people receive hospital care for injuries each year.
Older (49+ years)	- 6% of older people reported abuse in the past month.

**Table 2:** (**A**). Study characteristics. (**B**). Outcomes and key findings.

(A)						
Study	Year	Country	Design	Population	Sample Size	IPV Type/Violence Assessed
Cabrales-Tejeda et al. [18]	2023	Mexico	Prospective cross-sectional	Women attending gynecology clinic	325	Physical, psychological, sexual, emotional IPV
Kishton et al. [19]	2022	USA	Retrospective cohort (claims data)	Women with IPV-related insurance claims	10,980	Documented experience of violence (DEV)
Ghafournia & Healey [20]	2022	Australia	Retrospective cross-sectional	Women presenting to regional ED	161	Domestic violence & sexual assault
Baker et al. [21]	2021	USA	Cross-sectional survey	Incarcerated women in trauma therapy	115	Interpersonal & non-interpersonal trauma
Charak et al. [22]	2023	UK	Cross-sectional (latent class analysis)	Trauma-exposed national adult panel	1091	Multiple interpersonal violence forms
Daugherty et al. [23]	2022	USA	Cross-sectional survey	Female IPV survivors (shelters/online)	93	Intimate partner violence
Ford-Gilboe et al. [24]	2020	Canada	RCT	Women experiencing IPV (past 6 months)	462	IPV (any type)
Guiguet-Auclair et al. [25]	2022	France	Multicenter case–control	Women in relationships ≥ 12 months	361	IPV (validated WAST)
Bentley et al. [26]	2022	Spain	Cross-sectional	Migrant women using support services	563	Physical, sexual, psychological IPV
Chaquila et al. [27]	2023	Peru	Cross-sectional national analysis	Women aged 15–49	18,621	Physical, sexual, emotional IPV
Agde et al. [28]	2024	Ethiopia	RCT	Pregnant women & husbands (rural)	864	Physical, psychological, sexual IPV
Barata et al. [29]	2025	Canada	RCT (SARE/EAAA)	First-year university women	153 women/206 relationships	Emotional, physical, severe IPV, harassment
Crespo et al. [30]	2025	Spain	RCT (secondary analysis)	Survivors receiving trauma-focused CBT	148	Psychological, physical, sexual, economic IPV
Gibson et al. [31]	2020	Ethiopia	Randomization experiment	Rural adults (men & women)	809	Attitudes toward physical IPV
Kelly et al. [32]	2024	South Africa	Longitudinal cohort	Adolescent girls & young women	2183	Physical IPV
Mahapatro et al. [33]	2024	India	RCT	Pregnant married women	211	Physical, psychological, sexual IPV
Panjaphothiwat et al. [34]	2025	Thailand	Cross-sectional	Pregnant women (COVID-19 period)	496	Psychological, physical, sexual IPV
Taft et al. [35]	2025	Australia	Cluster RCT	Adult women in general practice	45,438	Physical, emotional, psychological, sexual, financial IPV
**(B)**						
**Study**	**IPV Prevalence/Key Outcomes**		**Assessment Method**	**Mental Health Outcomes**	**Risk Factors/Other Findings**	**Limitations**
Cabrales-Tejeda et al. [18]	IPV ever: 52%		No standardized mental health instrument used; study focused on IPV prevalence and associated risk factors	—	Childhood abuse, jealousy, substance use, economic stress	Single site; self-report
Kishton et al. [19]	Higher ER (aOR 2.6) & inpatient use (aOR 2.2)		Administrative diagnostic codes for mental health and substance use disorders derived from insurance claims data	↑ MH & substance use diagnoses	Increased healthcare costs	Insurance-based; coding limits
Ghafournia & Healey [20]	>50% recurrent ED visits		Clinical symptom documentation from emergency department records; no validated psychometric scales specified	Strong link with MH symptoms	Pregnancy and Indigenous status high-risk	Small sample; single ED
Baker et al. [21]	High cumulative trauma exposure		Validated self-report psychometric scales assessing depression, PTSD, distress tolerance, guilt, and shame (battery of standardized trauma-related instruments)	↑ Depression, PTSD, anxiety	Interpersonal trauma predictive	Prison sample; recall bias
Charak et al. [22]	Polyvictimization: high IPV class		Life Events Checklist for trauma exposure and standardized measures of depression, anxiety, and DSM-5 PTSD symptoms, including diagnostic classification	↑ Depression (9×), anxiety (12×), PTSD (33×)	Victimization clustering	Self-report; cross-sectional
Daugherty et al. [23]	High abuse severity		PHQ-9 (depression), PCL-5 (PTSD), GAD-7 (anxiety), and Neuro-QOL Executive Function scale	↓ Executive functioning	PTSD and abuse severity predict EF impairment	Small; no objective testing
Ford-Gilboe et al. [24]	Longitudinal improvements in IPV-related symptoms		Validated depression and PTSD symptom scales assessed longitudinally at baseline, 3, 6, and 12 months	↓ Depression and PTSD over 12 months	Tailored intervention helps severe cases	No true control group
Guiguet-Auclair et al. [25]	WAST AUC: 0.99; sensitivity 97.7%		Women Abuse Screening Tool (WAST) only; mental health outcomes not assessed	—	Self-administered preferred	Some sampling bias
Bentley et al. [26]	IPV: 78%		PHQ-9 (depression) and GAD-7 (anxiety) in a large population-based migrant sample	Depression, PTSD associated	Psychological + comorbid IPV highest burden	Support-seeking bias
Chaquila et al. [27]	IPV: 15.4%		PHQ-9 (depressive symptoms) derived from nationally validated ENDES survey data	Depressive symptoms: 27.1%	Wealth modifies IPV-depression link	No childhood abuse data
Agde et al. [28]	IPV knowledge > 50%		None—IPV knowledge/attitudes measured via adapted structured questionnaire; no validated mental health instrument used	—	Education, antenatal care protective	Baseline cross-section only
Barata et al. [29]	54% reduction in IPV (intervention)		Not assessed; intervention study evaluating IPV reduction following a sexual assault resistance program (EAAA)	—	Effects extended to emotional & physical IPV	Single IPV measurement
Crespo et al. [30]	High dropout (45%)		PTSD, depression, anxiety assessed using EGEP-5, BDI-II, BAI, PANAS, DERS	↑ Anxiety predicts dropout	Employment instability; recent IPV	Single therapist; small sample
Gibson et al. [31]	IPV acceptance: 18% (direct) vs. 28% (indirect)		Attitudes toward IPV assessed via list randomization experiment	—	Low education, male-controlled finances ↑ acceptance	Cultural sensitivity issues
Kelly et al. [32]	High-risk trajectory: 26.7%		Perceived stress (Cohen Stress Scale) and biomarkers	↑ CRP inflammatory biomarker	Cash transfers buffer stress–IPV link	Stress measured once
Mahapatro et al. [33]	Severe IPV ↓ by 90% post-intervention		Quality of life via SF-36; DV via AAST	↑ QoL across domains	Slight improvements in RCH outcomes	No follow-up; self-report
Panjaphothiwat et al. [34]	IPV: 15.5%		Mental health inferred via validated DV questionnaire (Cronbach α ≈ 0.70); no diagnostic tools	↑ Depression	Low income, unintended pregnancy, alcohol use	Underreporting likely
Taft et al. [35]	IPV identified: 0.58%		Mental health inferred from GP records and algorithm-based DVA identification	—	South Asian women under-identified	Coding limitations; no consent

Glossary: ↑: increased, ↓: decreased; DV: Domestic Violence; DEV: Documented Experience of Violence; ED: Emergency Department; IPV: Intimate Partner Violence; PTSD: Post-Traumatic Stress Disorder;; AUC: Area Under the Curve.

**Table 3:** Risk of bias assessment for the included studies.

Study	Design	RoB Tool Used	Overall RoB	Key Concerns
Cabrales-Tejeda et al. [18]	Prospective, analytical cross-sectional, single tertiary gynecology clinic	JBI—Analytical Cross-sectional	Some concerns/moderate (3)	Convenience sample from one hospital; reliance on self-reported IPV; limited adjustment for confounders; generalizability restricted to similar clinical settings.
Kishton et al. [19]	Retrospective cohort using private insurance claims	ROBINS-I	Some concerns/moderate (3)	Selection restricted to privately insured women; IPV identified through ICD codes (possible misclassification); residual confounding likely despite adjustments; good internal consistency of claims data.
Ghafournia & Healey [20]	Retrospective cross-sectional, single regional ED	JBI—Analytical cross-sectional	Some concerns/moderate (3)	Single emergency department; relatively small sample; potential under-reporting of IPV/SA; limited exploration of non-attenders; outcome and exposure based on routine documentation and self-report.
Baker et al. [21]	Cross-sectional survey among incarcerated women	JBI—Analytical cross-sectional	High/serious (4)	Highly selected, treatment-seeking prison sample; trauma histories measured retrospectively with limited event detail; frequency and timing of trauma not captured; multiple unmeasured confounders (e.g., lifetime psychiatric comorbidity).
Charak et al. [22]	Cross-sectional latent class analysis in national trauma-exposed panel	JBI—Analytical cross-sectional	Some concerns/moderate (3)	Online panel may exclude non-digital or marginalized groups; self-reported victimization and mental health; cross-sectional design precludes causal inference; no detailed racial/ethnic data.
Daugherty et al. [23]	Cross-sectional survey of IPV survivors (shelters and online)	JBI—Analytical cross-sectional	High/serious (4)	Small sample; recruitment from shelters and online platforms (strong selection bias); perceived executive functioning and symptoms based solely on self-report; no objective neuropsychological testing; cross-sectional design.
Ford-Gilboe et al. [24]	RCT of online safety and health intervention for women experiencing IPV	RoB 2	Some concerns (2)	Lack of a “no-intervention” control (comparison of tailored vs. non-tailored versions); self-selected online, English-speaking sample; outcomes based on self-reported symptoms; possible “survey-as-intervention” effect.
Guiguet-Auclair et al. [25]	Multicenter case–control validation of the French WAST	JBI—Case–control checklist	Low (1)	Clear inclusion criteria; appropriate measurement of exposure (WAST) and outcome (IPV status); excellent psychometric performance; some sampling bias in controls (partly recruited from investigators’ circles) but unlikely to substantially affect internal validity.
Bentley et al. [26]	Cross-sectional, observational study of migrant women in Spain	JBI—Analytical cross-sectional	Some concerns/moderate (3)	Support-seeking migrant sample (selection bias); reliance on self-reported IPV and mental health; cross-sectional analysis limits causal interpretation; limited adjustment for contextual migration-related factors.
Chaquila et al. [27]	Cross-sectional analysis of national survey of Peruvian women	JBI—Analytical cross-sectional	Some concerns/moderate (3)	IPV and depressive symptoms measured via self-report; cross-sectional design; no data on lifetime or childhood abuse; residual confounding by unmeasured social factors; large, nationally representative sample strengthens external validity.
Agde et al. [28]	Cluster RCT in rural Ethiopia (baseline knowledge and attitudes)	RoB 2	Some concerns (2)	Baseline analysis essentially cross-sectional; self-reported attitudes and knowledge (social desirability bias); limited comparable literature for men; randomization process described but clustering and contextual confounding remain possible.
Barata et al. [29]	RCT (SARE/EAAA sexual assault resistance program) with IPV outcomes in first-year university women	RoB 2	Low (1)	Robust randomized design; PV outcomes measured prospectively over 12 months; some limitations due to small IPV substudy, single measurement of IPV, and limited diversity (mostly white, heterosexual students) but low risk of major internal bias.
Crespo et al. [30]	RCT (secondary analysis) of trauma-focused CBT for survivors of IPVAW	RoB 2	High/serious (4)	High overall dropout (45%), especially in first sessions; small sample size; single therapist delivering both conditions; reasons for dropout partly self-reported and prone to bias; limited power to detect differences between treatment arms.
Gibson et al. [31]	Randomization experiment (list experiment) on attitudes towards wife-beating in rural Ethiopia	RoB 2	Low (1)	Random assignment to direct vs. indirect questioning; appropriate handling of list experiment; main limitation is small size of some subgroups and cultural sensitivity of topic; overall internal validity is strong for the main attitudinal outcome.
Kelly et al. [32]	Longitudinal cohort of adolescent girls and young women in South Africa	ROBINS-I	Some concerns/moderate (3)	Physical IPV measured repeatedly via self-report; sparse data in older ages; some biomarkers assessed only once; possible residual confounding despite detailed data; large sample and longitudinal design strengthen temporal inference.
Mahapatro et al. [33]	RCT of behavioral intervention package for pregnant women experiencing IPV	RoB 2	Some concerns (2)	Randomization described; outcomes based on self-reported IPV severity and QoL; only post-intervention measurement (no long-term follow-up); no partner data; conducted in one public tertiary hospital among low-income women, limiting generalizability.
Panjaphothiwat et al. [34]	Cross-sectional descriptive study of IPV in pregnant women during COVID-19	JBI—Analytical cross-sectional	Some concerns/moderate (3)	Self-reported IPV in antenatal setting; cross-sectional design; under-reporting likely due to stigma; limited exploration of non-attenders; nonetheless, sampling frame and measurement are described.
Taft et al. [35]	Cluster RCT in Australian general practice (baseline IPV identification data)	RoB 2	Some concerns (2)	Baseline data derived from routine electronic medical records; algorithm-based identification likely underdetects IPV; no individual patient consent; limited information on completeness and accuracy of EMR coding; clustering and ethnic disparities in recording acknowledged.

**Table 4:** Key findings stratified by risk of bias.

Outcome Domain	Low or Moderate Risk Studies	High-Risk Studies	Interpretation
IPV prevalence (population and clinical samples)	Chaquila et al. [27] (moderate); Cabrales-Tejeda et al. [18] (moderate); Kishton et al. [19] (moderate); Guiguet-Auclair et al. [25] (low)	Baker et al. [21] (high); Crespo et al. [30] (high)	Robust conclusion: IPV is common across populations. Exploratory: extremely high prevalence reflects selection of extreme-risk groups
Depression associated with IPV	Charak et al. [22] (moderate); Bentley et al. [26] (moderate); Chaquila et al. [27] (moderate); Ford-Gilboe et al. [24] (moderate)	Baker et al. [21] (high)	Robust conclusion: IPV–depression association consistent across designs
PTSD associated with IPV	Charak et al. [22] (moderate); Bentley et al. [26] (moderate); Ford-Gilboe et al. [24] (moderate)	Baker et al. [21] (high)	Robust conclusion: IPV is a strong risk factor for PTSD. Exploratory: near-universal PTSD in incarcerated samples
Anxiety and suicidality	Charak et al. [22] (moderate); Crespo et al. [30] (high) (partial)	Baker et al. [21] (high)	Moderately robust: anxiety association supported; suicidality severity mainly from high-risk samples
Cognitive/executive dysfunction	—	Daugherty et al. [23] (high)	Exploratory only: hypothesis-generating evidence
Healthcare utilization and costs	Kishton et al. [19] (moderate); Taft et al. [35] (moderate)	Ghafournia & Healey [20] (moderate) (small sample)	Robust conclusion: IPV associated with increased healthcare utilization and under-detection
Biological stress markers	Kelly et al. [32] (moderate)	—	Moderately robust: supported by longitudinal design, limited replication
Effectiveness of interventions	Barata et al. [29] (low); Mahapatro et al. [33] (moderate); Ford-Gilboe et al. [24] (moderate)	Crespo et al. [30] (high)	Robust conclusion: interventions reduce IPV-related harm
Screening and identification of IPV	Guiguet-Auclair et al. [25] (low); Taft et al. [35] (moderate)	—	Robust conclusion: validated tools perform well; IPV remains under-identified

## Data Availability

No new data were created or analyzed in this study.

## Supplementary Materials

The following supporting information can be downloaded at: https://www.mdpi.com/article/10.3390/healthcare14040465/s1, Table S1: Frequency Table—region, Table S2: Frequency Table—countries, Table S3: Frequency Table—Studies, Table S4: Notes, Table S5: Statistics—Age, Table S6 A and B: Case processing summary and percentiles, Table S7: Variables according to study, Figure S1: Bayesian estimation.

## Abbreviations

The following abbreviations are used in this manuscript:

AUC	Area Under the Curve
DV	Domestic Violence
DEV	Documented Experience of Violence
ED	Emergency Department
FGM	Female Genital Mutilation
IPV	Intimate Partner Violence
MeSH	Medical Subject Headings
PCL-C	the PTSD Checklist-Civilian Version
PHQ-9	Patient Health Questionnaire-9
PTSD	Post-Traumatic Stress Disorder
WHO	World Health Organization
