# Supplementary material for: Invisible Wounds: A Systematic Review of Domestic Violence Against Women
Source: Healthcare (Basel). 2026 Feb 12;14(4):465. doi: 10.3390/healthcare14040465 (PMC12940249; doi:10.3390/healthcare14040465)
Supplement: Supplementary file 1 [file healthcare-14-00465-s001.zip › healthcare-4074539-supplementary.pdf]

Supplementary material

Table S1: Frequency Table - region

Frequency Table

Region

		Frequency	Percent	Valid Percent	Cumulative Percent
Valid	Africa	1	14.3	14.3	14.3
	Asia	1	14.3	14.3	28.6
	Europe	1	14.3	14.3	42.9
	Global / Multiregional	1	14.3	14.3	57.1
	Latin America	1	14.3	14.3	71.4
	North America	1	14.3	14.3	85.7
	Oceania	1	14.3	14.3	100.0
	Total	7	100.0	100.0	

Table S2: Frequency Table - countries

Countries

		Frequency	Percent	Valid Percent	Cumulative Percent
Valid	Australia	1	14.3	14.3	14.3
	India, Thailand, Ethiopia	1	14.3	14.3	28.6
	Mexico, Peru	1	14.3	14.3	42.9
	Multicountry dataset	1	14.3	14.3	57.1
	South Africa	1	14.3	14.3	71.4
	United Kingdom, France, Spain	1	14.3	14.3	85.7
	United States, Canada	1	14.3	14.3	100.0
	Total	7	100.0	100.0	

Table S3: Frequency Table - Studies

Study

		Frequency	Percent	Valid Percent	Cumulative Percent
Valid	Agde et al.	1	5.6	5.6	5.6
	Baker et al.	1	5.6	5.6	11.1
	Barata et al.	1	5.6	5.6	16.7
	Bentley et al.	1	5.6	5.6	22.2
	Cabrales-Tejeda et al.	1	5.6	5.6	27.8
	Chaquila et al.	1	5.6	5.6	33.3
	Charak et al.	1	5.6	5.6	38.9
	Crespo et al.	1	5.6	5.6	44.4
	Daugherty et al.	1	5.6	5.6	50.0
	Ford-Gilboe et al.	1	5.6	5.6	55.6
	Ghafournia & Healey	1	5.6	5.6	61.1
	Gibson et al.	1	5.6	5.6	66.7
	Guiguet-Auclair et al.	1	5.6	5.6	72.2
	Kelly et al.	1	5.6	5.6	77.8
	Kishton et al.	1	5.6	5.6	83.3
	Mahapatro et al.	1	5.6	5.6	88.9
	Panjaphothiwat et al.	1	5.6	5.6	94.4
	Taft et al.	1	5.6	5.6	100.0
	Total	18	100.0	100.0	

Table S4: Notes

Notes

		Frequency	Percent	Valid Percent	Cumulative Percent
Valid	Approx. midpoint of 25–49 age range, Peru national samp	1	5.6	5.6	5.6
	Approximate mean; GP records, Australia	1	5.6	5.6	11.1
	Estimated midpoint of adult sample (18–65)	1	5.6	5.6	16.7
	Mean; incarcerated women in U.S.	1	5.6	5.6	22.2
	Mean; IPV survivors, U.S. shelters	1	5.6	5.6	27.8
	Mean; migrant women in Spain	1	5.6	5.6	33.3
	Mean; pregnant women, India	1	5.6	5.6	38.9
	Mean; pregnant women, Thailand	1	5.6	5.6	44.4
	Mean; RCT, online IPV intervention, Canada	1	5.6	5.6	50.0
	Mean; validation of WAST tool, France	1	5.6	5.6	66.7
	Median (IQR 30–46); outpatient clinic in Mexico	1	5.6	5.6	72.2
	Median age for ED cohort in Australia	1	5.6	5.6	77.8
	Median; adolescent/young adult cohort in South Africa	1	5.6	5.6	83.3
	Median; trauma-focused CBT in Spain	1	5.6	5.6	88.9
	University women; mean participant age	1	5.6	5.6	94.4
	Women's mean age; Ethiopia RCT	1	5.6	5.6	100.0
	Total	18	100.0	100.0	

Table S5: Statistics - Age

Statistics

Age

N	Valid	18
	Missing	0
Mean		33.267
Median		35.450
Std. Deviation		7.9806
Range		32.6
Minimum		15.0
Maximum		47.6

Figure S1: Bayesian estimation

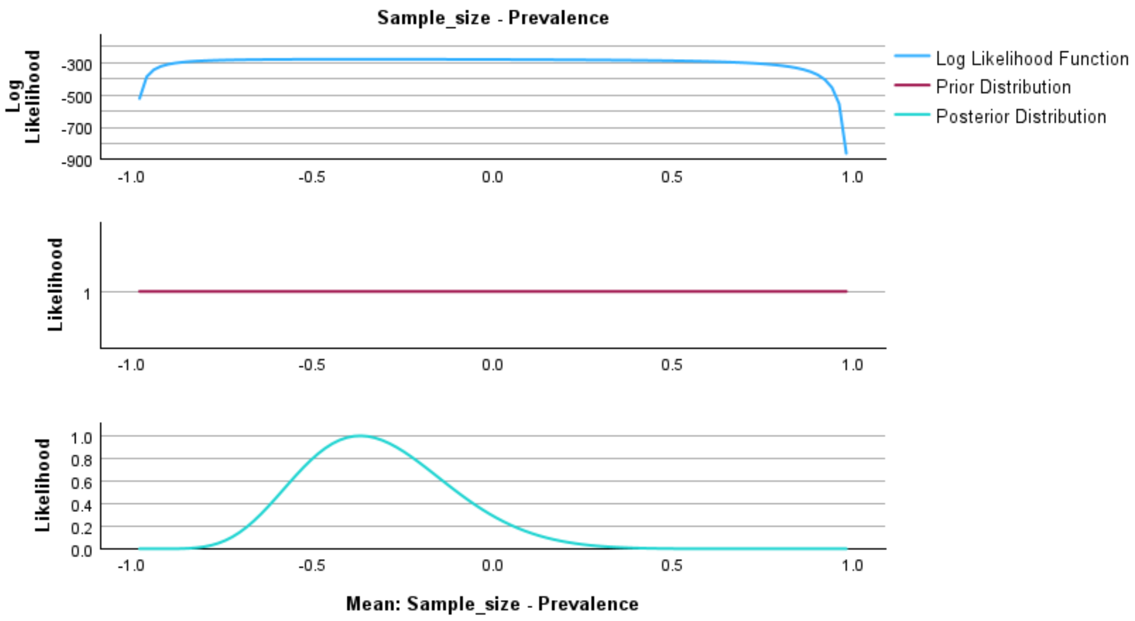

To illustrate the conceptual framework of Bayesian estimation we created a simple example showing how information from data and information from assumptions combine. In this example, the first curve stays almost flat, which means the data alone do not strongly point to any specific value for IPV prevalence. The second curve shows that we did not start with any assumptions about what the prevalence should be. The final curve shows how the information from all the studies in our review comes together to form our updated estimate of IPV prevalence.

Table S6 A and B: Case processing summary and percentiles

Total Sample

Case Processing Summary

	Cases					
	Valid		Missing		Total	
	N	Percent	N	Percent	N	Percent
Sample_size	18	100.0%	0	0.0%	18	100.0%
Prevalence	18	100.0%	0	0.0%	18	100.0%

Percentiles

		Percentiles		
		25	50	75
Weighted Average (Definition 1)	Sample_size	159.00	479.00	1364.00
	Prevalence	27.700	53.500	86.250
Tukey's Hinges	Sample_size	161.00	479.00	1091.00
	Prevalence	28.000	53.500	85.000

Binary matrix of psychological outcomes and healthcare indicators across included studies

Table S7: Variables according to study

Study	Depression	Anxiety	PTSD	Suicidality	Daily disfunction	Cost to healthcare	Use of insurance
Cabrales-Tejeda et al.	1	0	0	0	0	0	0
Kishton et al.	0	0	0	0	0	1	1
Ghafournia & Healey	1	0	0	0	0	0	0
Baker et al.	1	1	1	1	0	0	0
Charak et al.	1	1	1	1	0	0	0
Daugherty et al.	1	1	1	0	1	0	0
Ford-Gilboe et al.	1	1	1	0	0	0	0
Guiguet-Auclair et al.	0	0	0	0	0	0	0
Bentley et al.	1	1	1	0	0	0	0
Chaquila et al.	1	0	0	0	0	0	0
Agde et al.	0	0	0	0	0	0	0
Barata et al.	1	0	0	0	0	0	0
Crespo et al.	1	1	1	0	0	0	0
Gibson et al.	0	0	0	0	0	0	0
Kelly et al.	0	0	0	0	0	0	0
Mahapatro et al.	1	1	0	0	0	0	0
Panjaphothiwat et al.	1	1	0	0	0	0	0
Taft et al.	0	0	0	0	0	0	1
